# Decoding post-stroke motor function from structural brain imaging

**DOI:** 10.1016/j.nicl.2016.07.014

**Published:** 2016-08-02

**Authors:** Jane M. Rondina, Maurizio Filippone, Mark Girolami, Nick S. Ward

**Affiliations:** a Sobell Department of Motor Neuroscience, Institute of Neurology, University College London, UK; b Department of Data Science, EURECOM, France; c Department of Statistics, University of Warwick, UK

**Keywords:** Stroke, Motor impairment, Lesion patterns, Machine learning, Gaussian processes, Multiple kernel learning, Features extraction, Patterns of lesion probability, Lesion load

## Abstract

Clinical research based on neuroimaging data has benefited from machine learning methods, which have the ability to provide individualized predictions and to account for the interaction among units of information in the brain. Application of machine learning in structural imaging to investigate diseases that involve brain injury presents an additional challenge, especially in conditions like stroke, due to the high variability across patients regarding characteristics of the lesions. Extracting data from anatomical images in a way that translates brain damage information into features to be used as input to learning algorithms is still an open question. One of the most common approaches to capture regional information from brain injury is to obtain the lesion load per region (i.e. the proportion of voxels in anatomical structures that are considered to be damaged). However, no systematic evaluation has yet been performed to compare this approach with using patterns of voxels (i.e. considering each voxel as a single feature). In this paper we compared both approaches applying Gaussian Process Regression to decode motor scores in 50 chronic stroke patients based solely on data derived from structural MRI. For both approaches we compared different ways to delimit anatomical areas: regions of interest from an anatomical atlas, the corticospinal tract, a mask obtained from fMRI analysis with a motor task in healthy controls and regions selected using lesion-symptom mapping. Our analysis showed that extracting features through patterns of voxels that represent lesion probability produced better results than quantifying the lesion load per region. In particular, from the different ways to delimit anatomical areas compared, the best performance was obtained with a combination of a range of cortical and subcortical motor areas as well as the corticospinal tract. These results will inform the appropriate methodology for predicting long term motor outcomes from early post-stroke structural brain imaging.

## Highlights

• Accurate predictions of stroke outcome can contribute to improve clinical efficacy.
• Lesion load and patterns of voxels with different anatomical masks were compared.
• Gaussian Process Regression with different feature sets was used to decode outcome.
• Patterns of voxels resulted in more accurate predictions than lesion load.
• Features from motor areas and CST were the most informative for motor prediction.

## 1 Introduction

The ability to predict long term outcome after stroke is urgently required in order to facilitate a stratified approach to clinical decision making (Ward, 2015). It has long been known that information encoded in brain lesions (e.g. extent and location) can explain variability in post-stroke outcomes (Bayona et al., 2005, Geva et al., 2011, Särkämö et al., 2009, Schiemanck et al., 2005, Yang et al., 2008, Zhu et al., 2010), but no approaches have been routinely incorporated into clinical practice.

Machine learning (ML) techniques are potentially useful for clinical applications, aiming to provide sensitive and specific diagnostic and prognostic indicators for individuals, as opposed to analysing statistical group differences (Wang et al., 2010). In neuroimaging, clinical applications of ML methods have initially focused mainly on binary classification of disease states (Davatzikos et al., 2005, Teipel et al., 2007, Fu et al., 2008, Klöppel et al., 2008, Vemuri et al., 2010). More recently, decoding of outcomes represented by continuous scales has also become increasingly common in several neurological and psychiatric conditions through predictive multivariate regression methods (Cohen et al., 2011).

The extraction of features from brain images in a way that is meaningfully related to the clinical condition being studied is a fundamental step in a predictive analysis framework. In the context of stroke, feature extraction from structural neuroimaging is additionally challenging due to the high variability in anatomical location and extension of brain injury. Although lesion characteristics can potentially contribute towards making accurate predictions of the likely level of impairment and recovery, there is currently no consensus on how to quantify these characteristics.

Progress has been made in predicting language outcomes using features derived from stroke lesions (Payabvash et al., 2010; Hope et al., 2013, Hope et al., 2015) but predicting motor outcomes is lagging behind. One of the most common approaches to quantify characteristics from lesions is summarizing the proportion of voxels in each region of interest (ROI) that are considered to be part of a lesion. This information is commonly referred to as *lesion load* and it is obtained using anatomical masks to define the ROIs and a method to segment the lesions, either manually (Kim et al., 2014) or automatically (Hope et al., 2013, Hope et al., 2015). Recently, voxel-based lesion symptom mapping (VLSM, (Bates et al., 2003)) has also been proposed as a way to extract features from stroke lesions to be used as input to machine learning models. Voxel-based lesion symptom values are obtained for each voxel through a statistical test on the continuous scores representing the symptom between two groups (which are defined according to the presence or absence of a lesion in that particular voxel). The voxelwise maps resulting from this method are used as a way to define a mask to restrict voxels (Munsch et al., 2015) or to build symptom or condition specific ROIs (Forkert et al., 2015).

In this paper we have directly compared a range of approaches for assessing the relationship between structural brain damage and long term motor outcome in chronic stroke patients. Using structural MRI images from 50 patients we derived lesion probability images (i.e., images where each voxel is assigned a value between 0 and 1 representing the likelihood of being part of injured tissue). We wanted to investigate which features have the highest power to decode the individual level of motor impairment. There are two key questions: firstly, what type of data should be extracted from the images? Secondly, which are the key brain regions from which data should be extracted? To investigate the first question, we used two strategies to extract information from images: *i*) patterns of voxels, where each feature corresponds to a single voxel representing lesion probability, and *ii*) anatomical summarization, where each feature corresponds to the lesion load in an ROI. To investigate the second question, we employed a number of different approaches to define anatomical regions: *i*) regions of interest (ROIs) from an anatomical atlas; *ii*) a mask delimiting the corticospinal tract (CST); *iii*) combination of all ROIs and the CST; *iv*) a subset of the ROIs expected to be related to motor function; *v*) combination of the subset of ROIs and the CST; *vi*) active voxels from fMRI acquired with a motor task in healthy controls; *vii*) a mask restricting voxels to lesions; *viii*) voxels selected through lesion-symptom mapping. Additionally, we performed a secondary analysis, applying multiple kernel learning techniques using kernels extracted from brain regions to investigate the possibility of assessing the relevance of each anatomical pattern.

## 2 Material and methods

### 2.1 Study population

Fifty patients that had their first stroke at least three months before the collection of the data (mean 29.1, std 31.1 months) participated in the study. The patients had mean age 54.2 years (std 12.6), Seventeen patients were female and in 18 patients the right hand was affected. Complete demographic and clinical characteristics of each patient can be found in the Supplementary material (Table S1). The extent and location of the lesions for each patient (Fig. S1) is also presented. A control group was composed by 23 age-matched healthy subjects who reported no history of neurological or psychiatric illness, vascular disease or hypertension. All subjects provided full written consent in accordance with the Declaration of Helsinki. The study was approved by the Joint Ethics Committee of the UCL Institute of Neurology, The National Hospital for Neurology and Neurosurgery and UCL Hospitals NHS Foundation Trust.

#### 2.1.1 Motor scores

Measures of motor impairment in the contralesional upper limb were obtained using four different assessment scales: Action Research Arm Test (ARAT) (Lyle, 1981), grip strength (GS) (Sunderland et al., 1989), Motricity Index (MI) (Bohannon, 1999) and Nine-Hole Peg Test (NHPT) (Mathiowetz et al., 1985).

As the different motor scores are correlated but also complementary, a single representative measure was calculated using principal component analysis (PCA). Considering *Y* as a matrix of 50 examples and 4 labels (corresponding to the number of patients and motor scores, respectively), the PCA was obtained using the following steps:

1. Calculate the mean of each score across patients and subtract it from *Y* (*zero mean Y*);
2. Obtain the covariance matrix from *zero mean Y (cov zero mean Y)*;
3. Find the eigenvalues of *cov zero mean Y*.

A vector **y** = [*y*_1_, …, *y*_*m*_] where *m* is the number of subjects represents the first principal component (FPC) of the four scores, which accounts for the greatest possible variance across them. This approach has the advantage of avoiding floor and ceiling effects encountered with individual measures.

### 2.2 Images acquisition and pre-processing

T1-weighted high resolution magnetic resonance images were acquired using a 3 T Allegra system (Siemens AG, Erlangen, Germany) with the following protocol: number of slices = 176, slice thickness = 1 mm, matrix size = 224 × 256, in-plane resolution = 1 mm × 1 mm.

The origin of each image was set at the anterior commissure. Images from patients that had injury predominantly in the left hemisphere were flipped in relation to the mid-sagittal plane so that all scans presented lesion in the right hemisphere. Images from all subjects were segmented into grey matter, white matter, cerebrospinal fluid and then normalized using the New Segment routine in SPM8 (http://www.fil.ion.ucl.ac.uk/spm/).

Lesion probability images were obtained from the high-resolution T1-weighted volumetric MRI scans using an automatic method for detection of outlier voxels (Seghier et al., 2008). This method is based on the assumption that lesions are characterized as atypical voxels regarding expected brain tissues (grey matter, white matter and cerebrospinal fluid). The characterization of tissues uses the unified segmentation-normalization approach (Ashburner and Friston, 2005) modified to include an extra tissue to account for the perturbation introduced by lesions. Grey and white matter segmented tissues from patients are compared with the corresponding tissues from healthy control subjects in a voxel by voxel way. As a result, each voxel is represented by a value between 0 and 1 that quantifies the likelihood of it being part of injured tissue.

### 2.3 Segmentation of lesions

**Fig. 1:**
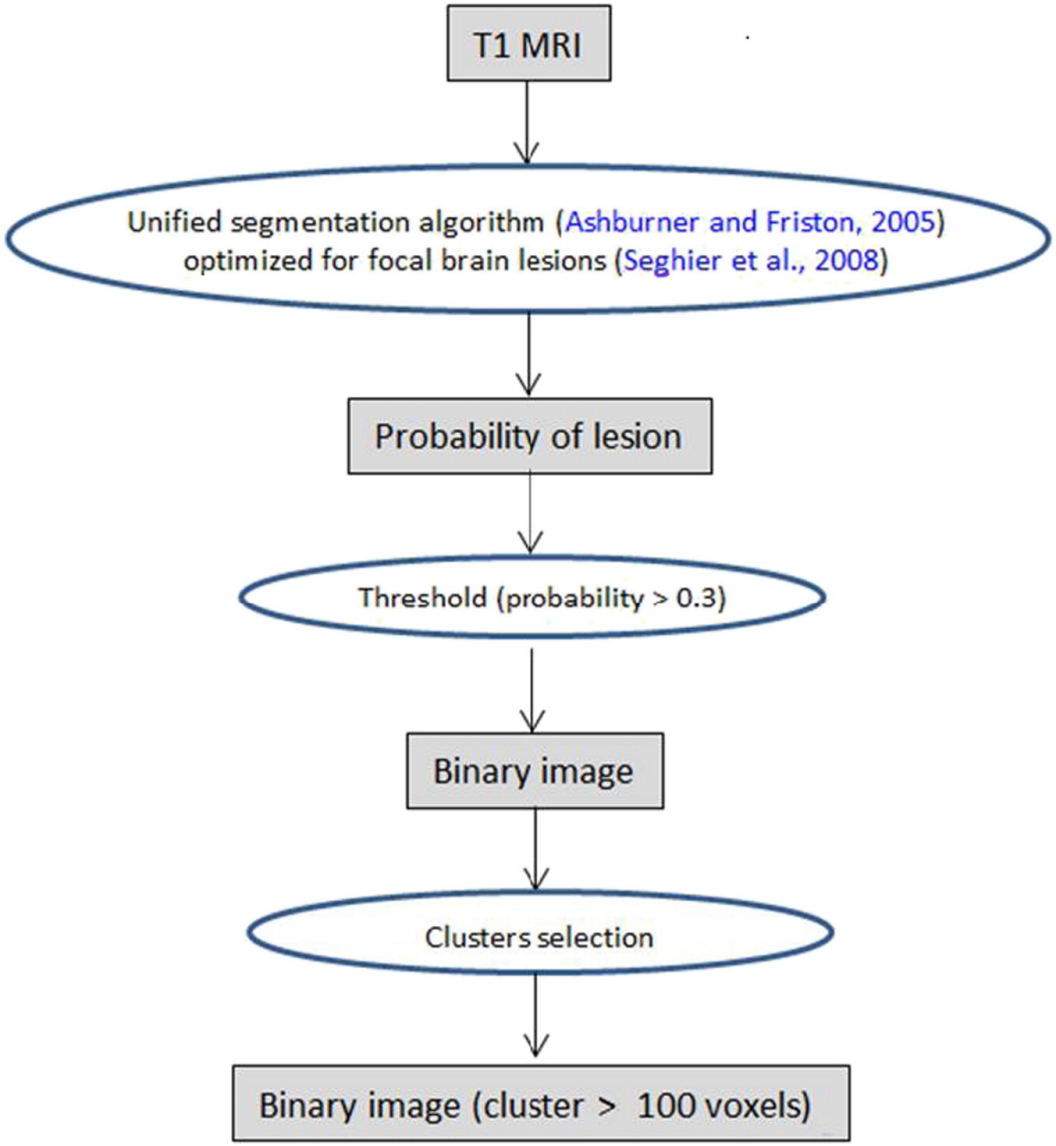
Steps for segmentation of lesions.

Fig. 1 presents the steps that were performed to obtain binary images of the lesions. Images representing lesion probability were derived from T1 anatomical images according with the procedure described in the previous section. In order to segment the lesions we applied a threshold selecting the voxels with probability of being part of injured tissue > 0.3, producing binary images. Finally we selected only contiguous clusters with 100 or more voxels. See (Seghier et al., 2008) for a detailed explanation regarding the rationale behind both parameters (threshold value and cluster size) and comparison with manually traced lesions. We also performed additional tests to check the adequacy of these parameters to segment lesions in our images (Supplementary material, item 2).

**Fig. 2:**
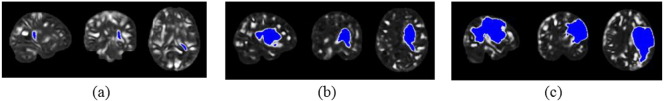
Examples of segmented lesions: (a) small; (b) medium; (c) large.

Fig. 2 shows examples of lesions segmented according to the described approach. The binary images corresponding to lesions (visualized in blue) were overlaid on the lesion probability images (visualized in grayscale).

**Fig. 3:**
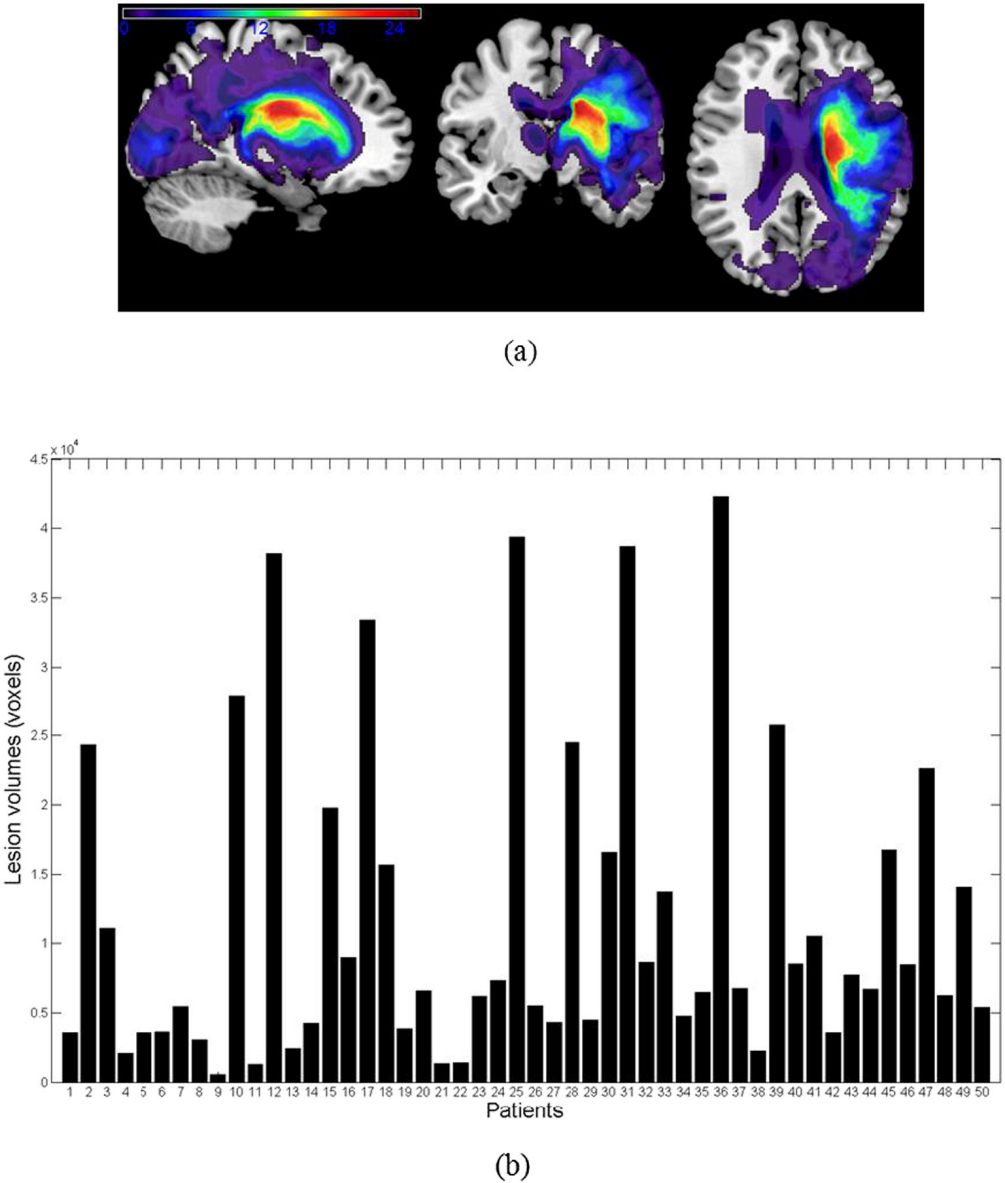
Lesion volumes per patient. (For interpretation of the references to colour in this figure, the reader is referred to the web version of this article.)

Fig. 3 (panel a) presents a map illustrating the overlap of segmented lesions obtained using our approach across all patients. The colour map represents the incidence of lesions in each voxel, ranging from purple (lesion in 1 subject only) to red (lesion in 26 out of the 50 subjects). Fig. 3 (panel b) presents a plot showing the volume of the segmented lesion for each patient according to the procedure described above. This plot illustrates the variability of the sample regarding the extent of the lesions across the patients. In the Supplementary material, we provide a figure that shows the distribution of the lesions in each patient across brain regions (Fig. S1).

### 2.4 Feature extraction

In the following sections we describe the strategies that we used to extract features. In the Section 2.4.1 we describe the approaches implemented to extract information from images of lesion probability (through patterns of voxels or lesion load). In the Section 2.4.2 we describe the approaches used to limit ROIs in the brain.

#### 2.4.1 Anatomical patterns and summarizations

##### 2.4.1.1 Patterns of voxels

Through this approach, each voxel inside a binary mask corresponds to a particular feature that represents lesion probability. It is important to note that independent of the strategy to delimit a mask, each voxel inside the delimited region corresponds to a particular feature in a pattern.

##### 2.4.1.2 Anatomical summarization (lesion load)

In this approach we used labelled masks to delimit ROIs and applied a summarization technique to transform the information encoded in each ROI into a single value. The summarization was based on the *lesion load*, which is the proportion of voxels in each region that were considered to be part of a lesion according to the segmentation algorithm described in the Section 2.3. Each summarization unit corresponds to a single feature.

**Fig. 4:**
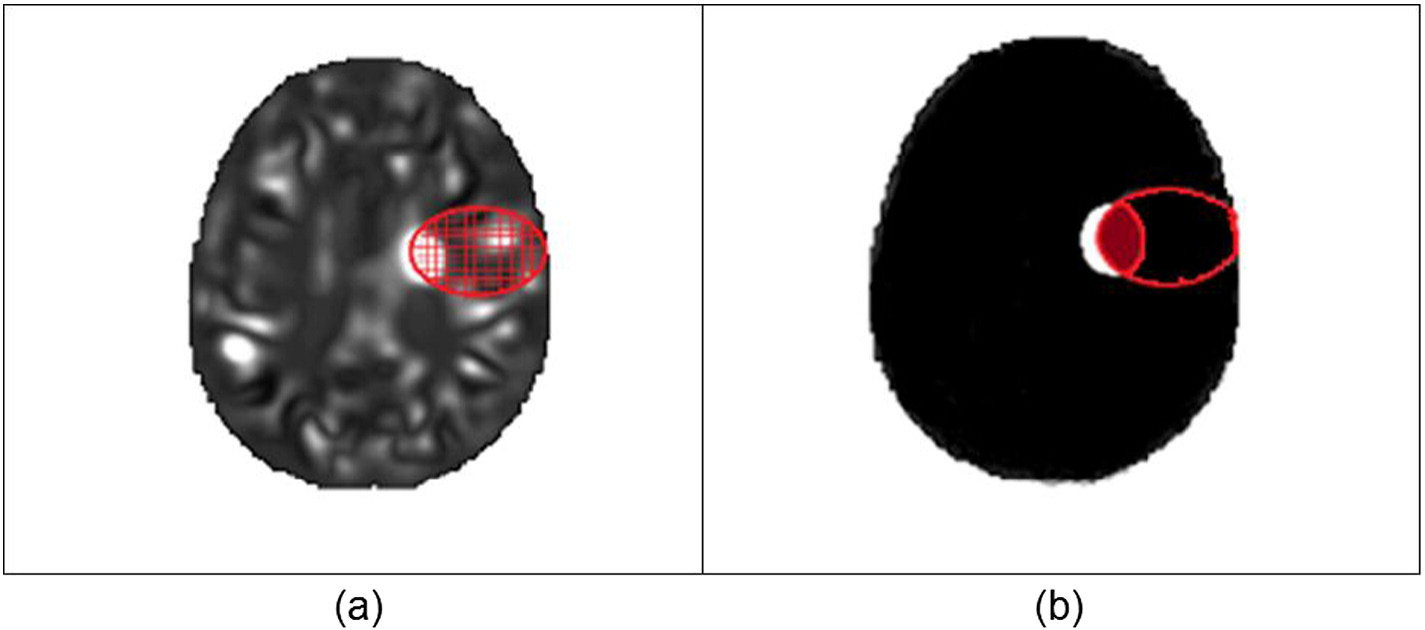
Extraction of features through patterns of voxels representing lesion probability (a) and anatomical summarization (lesion load) (b). (For interpretation of the references to colour in this figure, the reader is referred to the web version of this article.)

Fig. 4 illustrates the difference between the approaches to extract features. The area circled in red represents a mask corresponding to an ROI. The grid in panel (a) indicates that the mask encompasses a set of voxels each with a probabilistic value of lesion likelihood. In panel (b) the white region represents a lesion. The intersection between the lesion and the ROI divided by the size of the ROI corresponds to the lesion load, which is a single feature (as opposed to several features in the former approach).

#### 2.4.2 Approaches to delimit regions of interest

##### 2.4.2.1 Atlas-based ROIs

**Fig. 5:**
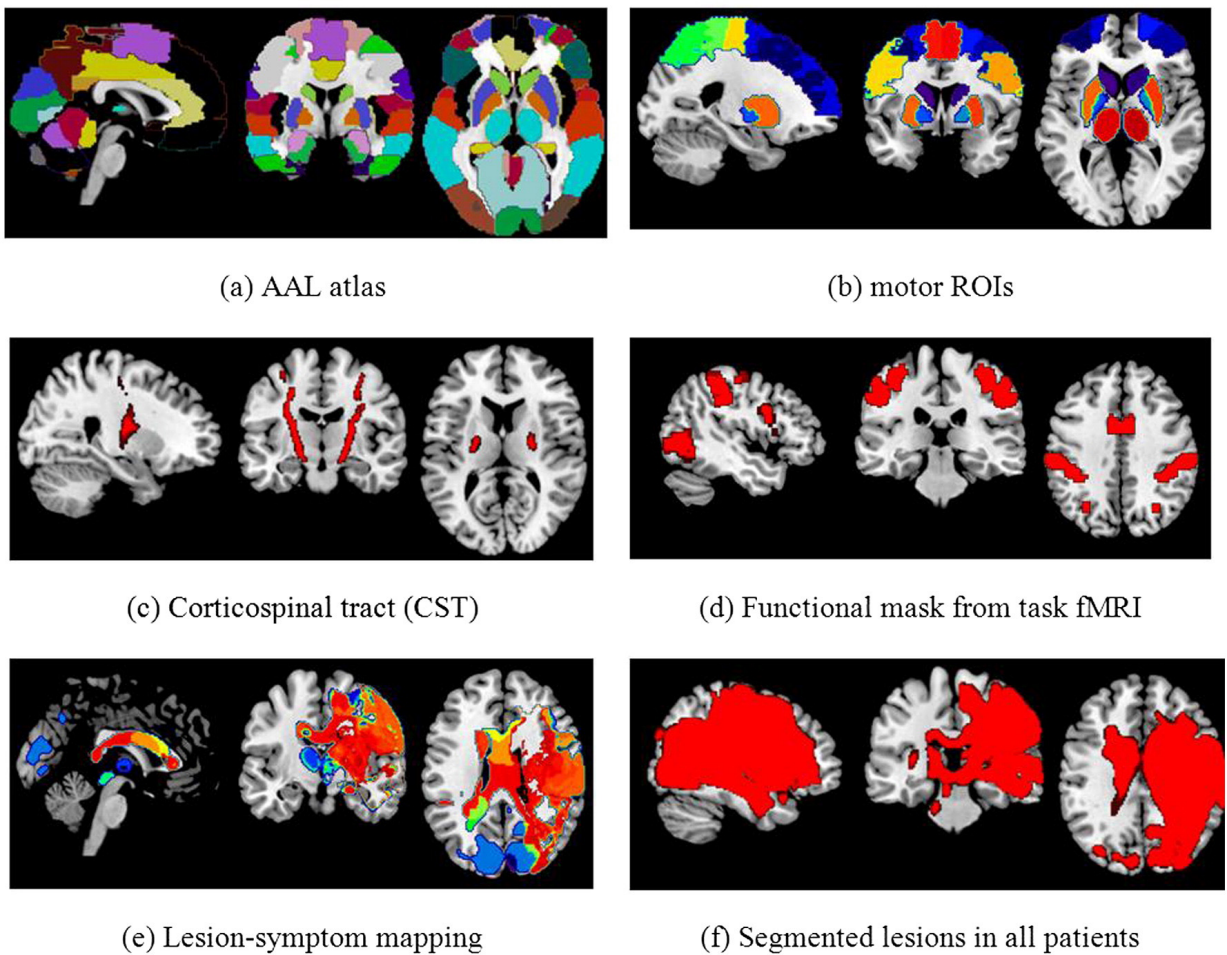
Delimitation of regions of interest.

Regions of interest were obtained automatically from the AAL atlas (Tzourio-Mazoyer et al., 2002), which comprises 116 separate regions corresponding to cortical and subcortical anatomical structures. Fig. 5 (panel a) illustrates the atlas overlapped on a structural brain template.

##### 2.4.2.2 Motor ROIs

We hypothesized that some brain regions might be more relevant than others for motor function. Thus we selected a subset of ROIs from the AAL atlas that correspond to regions expected to be related to motor and sensory function according to literature (Alexander et al., 1990, Kim et al., 1993, Cao et al., 1998, Hauk et al., 2004). The regions (illustrated in the Fig. 5, panel b) are the following: Postcentral gyrus, Precentral gyrus, Supplementary motor area, Superior frontal gyrus, Middle frontal gyrus, Inferior and Superior parietal regions, Thalamus, Caudate, Putamen and Pallidum.

##### 2.4.2.3 Corticospinal tract (CST)

Recent studies have proposed that the CST plays a relevant role in recovery of motor function post-stroke (Byblow et al., 2015, Stinear, 2010, Zarahn et al., 2011).

A CST mask was obtained by probabilistic tractography from nine age-matched healthy volunteers in a previous study (see details in Ward et al., 2008, Schulz et al., 2012). The mask is illustrated in Fig. 5 (panel c).

##### 2.4.2.4 Functional ROIs

With this mask we aimed to evaluate the effect of lesioned tissue in both the ipsilesional and contralesional hemispheres in areas involved in normal motor function. The voxels were selected through the application of General Linear Model (GLM) to fMRI data from healthy controls performing a motor task (hand grip using the dominant hand). Fig. 5 (panel d) illustrates the functional mask obtained from the contrast grasp versus rest (with family-wise error control *p* < 0.005, cluster size = 100 voxels). The mask was mirrored so that both hemispheres were considered, as in all other approaches. It is important to observe that the mask was obtained from healthy controls, so concerns are not applicable to the effect of lesion side and dominant hand in activation.

##### 2.4.2.5 Lesion-symptom mapping ROIs

Another way of obtaining ROIs that represent the relation between structure and function is through voxel-based lesion-symptom map (VLSM) (Bates et al., 2003, Forkert et al., 2015). A mask can be obtained by applying *t*-test in each voxel to compare the average of the motor score between the subjects that have a lesion and those who do not have a lesion in that particular voxel.

We implemented a variation on the approach proposed by Forkert et al. 2015. The authors built problem-specific ROIs assigning labels to voxels according to the difference in the median modified Rankin Scale (mRS) of each group. The mRS is a commonly used scale for measuring the degree of disability or dependence in the daily activities of people who have suffered neurological disability (Bonita and Beaglehole, 1988). It varies from 0 (meaning perfect health without symptoms) to 6 (meaning death). They produced four problem-specific ROIs: voxels with median mRS difference d > 2 corresponding to the first ROI, voxels with 1 < d < 2 corresponding to the second ROI, voxels with 0 < d < 1 corresponding to the third ROI and all the remaining voxels within the brain corresponding to the fourth ROI. As our scale (the first principal component of the motor scores) is continuous with values ranging from − 2.0237 to 1.5815, we converted them to a discreet range, rounding each fractional value to the closest integer number (− 2, − 1, 0, 1 and 2). For each voxel we obtained the median of the rounded score of each subject that had lesion in that particular voxel. This procedure resulted in 5 ROIs illustrated in the Fig. 5 (panel e).

##### 2.4.2.6 Lesion-bounded ROI

For completeness, we also defined a mask considering only voxels that were part of a lesion in at least one patient (Fig. 5, panel f).

### 2.5 Prediction analysis

Data for each analysis can be represented by a matrix *X*, where each column corresponds to a particular feature and each row contains the set of features corresponding to a particular example (in our case, data derived from the structural image of a single patient).

The data matrices were standardized in the following way: each feature vector (row) was normalized by the Euclidean distance. Then each feature was scaled to zero mean and unit variance. A linear function (dot product) was used to build a covariance matrix *C*, with dimensions corresponding to the number of examples (50 × 50). Covariance is a measure of how much two variables change together, and the covariance function (or kernel) describes the spatial covariance of a random variable.

(1) $$C=X\;X^{T}$$

We applied Gaussian Process Regression (GPR) (Filippone et al., 2012, Marquand et al., 2010, Rasmussen, 2006) using the covariance matrices to predict the representative score for motor impairment (first principal component from ARAT, Grip, Motricity index and NHPT). The score was also standardized according to the same procedure applied to the features.

A Gaussian process (GP) is formally defined as a set of random variables such that any subset of them is jointly Gaussian. These Gaussian distributions need the definition of a mean and a covariance, and this is typically done by associating each of the GP random variables with elements of some input domain, say *R*^*d*^, and by defining mean and covariance as functions of the input domain. For instance, a covariance function that makes random variables highly correlated when the corresponding inputs are close by, makes the GP random variables smoothly change over the input domain. Different covariance functions determine different behaviours of these random variables over the input domain, suggesting that GPs can be interpreted as a distribution over functions.

Gaussian Process Regression uses GPs as priors over functions, and attempts to learn a posterior distribution over these functions after data are observed using Bayesian inference techniques. In GP regression, the assumption is that data are Gaussian distributed around an unobserved function *f(x)* that is modelled using GPs, namely *y* *=* *f(x)* *+* *ɛ*, with *ɛ* *∼* *N(0,σn2)*. GP regression can be seen as an extension of standard linear regression, where the unobserved functions are assumed to be linear combinations of the features *f(x)* *=* *x*^*T*^*w*. In GP regression, obtaining the posterior distribution over the unobserved function *f(x,)* and learning any parameter of the covariance function (e.g., the smoothness from data), requires standard algebraic operations involving the covariance matrix, that is the matrix of all pairwise covariances of the random variables at the inputs where observations are available.

In order to check whether the results obtained with GPR would be similarly true for another predictive multivariate regression method, we repeated all analyses using Support Vector Regression (SVR) (Drucker et al., 1997, Smola et al., 2004) with the same linear kernel.

#### 2.5.1 Learning from multiple sources

As a secondary analysis, we wanted to investigate the relevance of each ROI based on patterns of voxels representing lesion probability, following a similar procedure as in Filippone et al. 2012.

Given the covariance matrices *C*_*i*_ associated to *M* sources of information, it is possible to build the covariance of the Gaussian process in the following way:

(2) $$K=\sum_{i}^{M}C_{i\;}w_{i}$$

Considering that the covariance matrices were obtained by scaling all the sources in the same way it is possible to interpret the weights as the relevance of the associated sources.

The weights *w*_*i*_ were optimized by the standard multivariate Gaussian log-likelihood:

(3) $$L=-\frac{1}{2}\log\left|K\right|-\frac{1}{2}\;y^{T}K^{-1}y+\mathrm{const}$$

Multiple kernel learning (MKL) can be used in different ways (Gönen and Alpaydın, 2011). Different kernels (covariance matrices) can correspond to different notions of similarity (e.g. linear, polynomial, Gaussian, exponential) or to different representations of data (sources). In neuroimaging, different data sources may comprise different imaging modalities (e.g., T1-MRI or DWI-MRI), different ways of extracting data from a same modality (e.g., volumetric or cortical thickness in structural MRI; ADC in DWI), or different subsets of features. In this study we applied the latter approach using anatomical criteria to define the subsets of features from patterns of voxels. Thus the number of sources *M* was 118, corresponding to 116 regions from the AAL atlas and the CST divided into left and right portions (to be consistent with the structures from the atlas, which are unilateral).

### 2.6 Results evaluation

To assess how the results of the analysis generalize to an independent data set we used cross-validation. One round of cross-validation involves partitioning the data sample into disjoint subsets of examples, performing the analysis on one subset (the training set), and validating the analysis on the other subset (the validation or testing set). To reduce the variability, multiple rounds of cross-validation are performed using different partitions, and the results are averaged over the rounds.

In this study, we implemented a 10-fold cross-validation, which involves separating 10% of the examples from the original sample for test while the remaining examples are used for training. This splitting is repeated so that each example in the sample is used once for validation. After repeating this process (leaving out all examples), the final accuracy is quantified as the average of accuracies obtained across all 10 folds. It is important to note that all additional operations that involve data from different examples (as scaling labels, calculating the principal component of the motor scores and obtaining problem-specific ROIs) were performed within the cross-validation folds without using any information from the test data, thus ensuring the validity of the reported performance scores.

The measure of accuracy in each analysis was obtained through the correlation between real and predicted labels. Correlation values were obtained in each fold and the final measure (R) corresponds to the average across the folds. Additionally, we present the root mean squared error (RMSE) according to the following equation, where N is the number of examples per fold, *f*_*i*_ is the predicted label and *y*_*i*_ is the real label:

$$\mathrm{RMSE}=\sqrt{\frac{1}{N}\sum {\left(f_{i}-y_{i}\right)}^{2}}$$

To evaluate whether the difference in RMSE across folds resulting from different models is statistically significant we used Wilcoxon rank sum test.

## 3 Results

### 3.1 Learning from single sources: patterns of voxels representing lesion probability

In this section, we present results of GPR analyses using a single source (only one covariance matrix) for which each feature corresponds to a single voxel representing the probability of being atypical tissue. Results of all strategies to delimit ROIs as anatomical patterns are presented in Table 1. For each ROI we show the corresponding number of features (NF) used as input to the model (that corresponds to the number of voxels in the mask), the correlation between real and predicted labels (R) and the root mean squared error (RMSE). The measures of correlation and the RMSE resulted from the average of the folds in the cross-validation framework. We also present the result of the same analysis performed without any mask to delimit voxels (model M1, whole brain approach).

Limiting the analysis to voxels belonging to all ROIs from AAL atlas produced the same result as using all voxels in the whole brain (models M1 and M1.1: R = 0.72; RMSE = 0.73). A similar result (R = 0.73; RMSE = 0.72) was obtained when adding the CST to the AAL ROIs (model M1.3). Limiting the analysis to voxels in the CST (model M1.2) led to R = 0.65 (RMSE = 0.75). The accuracy increases to R = 0.80 (RMSE = 0.70) when limiting the analysis to the areas selected from the AAL atlas that are believed to be the more specifically related to motor function according to literature (model M1.4). The best prediction was obtained joining motor ROIs and CST (model M1.5: R = 0.83, RMSE = 0.68). The remaining masks - areas resulting from motor activation in fMRI (model M1.6), lesion-symptom mapping (model M1.7) and lesion-bounded (model M1.8) led to accuracies lower than the whole brain (respectively R = 0.67; RMSE = 0.78, R = 0.66; RMSE = 0.76, R = 0.65; RMSE = 0.76).

### 3.2 Learning from single sources: lesion load per regions

In this section we present results of GPR analyses for which each feature corresponds to the summarization of all voxels belonging to a particular region. The strategies to delimit regions are the same used in the approach described in the previous section. However, in this approach, labelled ROIs are used so that each feature is the lesion load in a particular region (i.e., the proportion of voxels in an ROI that are considered to be part of a lesion). Thus, the number of features corresponds to the number of regions. Table 2 presents the results of each strategy to define the ROIs.

Prediction based on the lesion load in the whole brain (i.e. the proportion of damage in the brain, model M2) resulted in R = 0.30 (RMSE = 0.94). When considering the lesion load in each of the 116 ROIs from the AAL atlas (model M2.1), the accuracy decreases to R = 0.20 (RMSE = 8.04). When adding the CST to the AAL ROIs (model M2.3), the accuracy increases to R = 0.25 (RMSE = 6.47). The best accuracy in this approach was obtained limiting the analysis to the CST alone (model M2.2: R = 0.51; RMSE = 0.84).

As opposed to the previous approach, prediction based on the lesion load in the motor ROIs did not improve the accuracy in relation to the whole brain (model 2.4: R = 0.21; RMSE = 1.10). However, it increases slightly when including the lesion load in the CST (model M2.5, R = 0.26; RMSE = 1.09). Predictions based on lesion load in the ROIs defined by lesion-symptom mapping and in the ROI based on lesions in at least one patient (models M2.7 and M2.8) produced results similar to the lesion load in the whole brain.

The extraction of features through patterns of voxels produced better results than extracting lesion load for all strategies used to delimit regions. According to the Wilcoxon rank sum test based on the root mean squared error (RMSE) across folds, the difference between categories was statistically significant when comparing the models involving all ROIs and CST (M1.1 with M2.1 and M1.3 with M2.3, *p* < 0.001) and when comparing the models restricting ROIs to motor regions (M1.4 with M2.4, *p* = 0.023) and motor regions + CST (M1.5 with M2.5, *p* = 0.018). Comparisons of analyses using different ROIs within the same approach were not statistically significant.

**Fig. 6:**
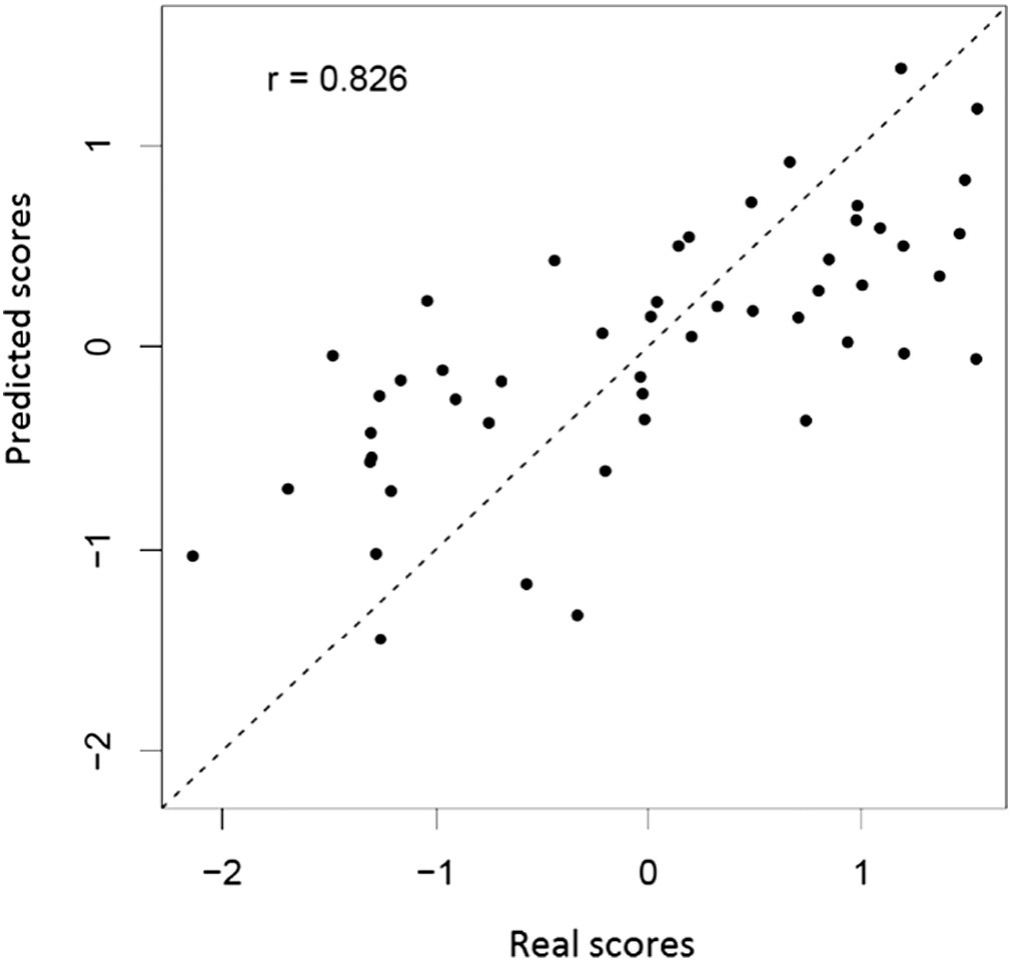
Prediction of first principal component of motor impairment scores based on voxels limited by motor ROIs and CST.

Fig. 6 shows a scatter plot of the real and predicted scores resulting from the model that produced the best accuracy (M1.5 - pattern of voxels limited by motor ROIs and CST).

We performed additional analyses using SVM with all the approaches to extract features. The results were well in line with the prediction derived from the GPR analyses, as the differences across strategies to define ROIs within each approach to extract features and the differences between the approaches remain similar to the differences described above. Please see the Tables S4 and S5 in the Section 3 of the Supplementary material for detailed results.

### 3.3 Learning from multiple sources

The correlation between actual and predicted scores using MKL was 0.66 (RMSE = 0.79). The Fig. S3 in the Supplementary material displays a plot of each ROI ranked in descending order of weight. The ROI corresponding to the highest weight (0.0267) was the right caudate nucleus, followed by the left inferior frontal gyrus, pars opercularis (weight 0.0264) and the right corticospinal tract (weight 0.0262). A table with the complete list of all 118 regions sorted by descending order of weights is also presented in the Supplementary material (item 4, Table S6). Note that this analysis is based on the maximisation of the log-marginal likelihood of the GPR approach, thus it does not give a full account of the uncertainty in this assessment. In a follow-up study we intend to carry out a deeper analysis of the uncertainty associated with these weights; the aim of this study was to gain some insights into the different ways to extract features and define regions of interest.

## 4 Discussion

We have applied machine learning techniques to ask how accurately upper limb motor impairment can be decoded from data derived from structural brain images in chronic stroke patients. We have compared a number of different approaches to extract data in the same subjects. There are two key findings: (i) Firstly, approaches that extract features through patterns of voxels produced better results than those extracting lesion loads per region; (ii) secondly, using only data from the corticospinal tract was not sufficient to produce the most accurate results.

For all approaches applied to define ROIs, the extraction of features corresponding to patterns of voxels produced higher accuracy in comparison to summarizing the information encoded in the voxels through lesion load per region. The differences between models using patterns of voxels and lesion load were statistically different when using all ROIs from AAL atlas, motor ROIs, combining AAL ROIs and CST and combining motor ROIs and CST. The best result was obtained using patterns of voxels from motor regions and CST together, suggesting that having some knowledge about the likelihood of damage to cortical regions (especially those thought to be motor related) may be important in accounting for variability in motor impairment.

Most studies investigating the anatomical correlates of motor outcomes after stroke have concentrated on quantifying damage in the corticospinal tract (Burke Quinlan et al., 2015, Byblow et al., 2015, Lindenberg et al., 2010, Schulz et al., 2012, Zhu et al., 2010). These studies have shown that damage in CST correlates well with motor outcomes. Our results are in alignment with these previous findings. While the information contained in the CST lesion load is less powerful than when represented as a pattern of voxels representing lesion probability, it is noteworthy that the lesion load in the CST presented higher predictive power than any other ROI compared. On the other hand, many of the findings in previous studies have concentrated on patients with subcortical infarcts. The patients in our study are diverse regarding both extension (Fig. 3) and location of lesions (Fig. S1, Supplementary material). Recent results suggest that predicting the motor behavioural consequences of stroke damage in more heterogeneous groups of patients is likely to require information about cortical as well as corticospinal tract damage (Park et al., 2016), and this is once again borne out by our findings (as results from models M1.4 and M1.5 suggest).

Another key point is that although damage to these motor regions (especially CST) can account for initial impairment, the anatomical correlates of subsequent recovery patterns are not yet known. It may be that survival of non-motor regions that are important for sensory processing, sustained attention, memory and learning have a key impact on recovery processes. The medium weights for predicting outcomes given to a variety of cortical regions in our MKL approach would be in keeping with this idea. However, the accuracy obtained through this method was lower than most of the approaches for learning from a single source using patterns of voxels. Thus further investigations would need to be carried out to investigate the potential of MKL to learn non-trivial combinations of features. Additional insights could be obtained with further investigations to select the most relevant data sources using some criterion to produce sparse results (i.e., assigning weight zero to some kernels during the optimization process).

It is important to note that although the way in which lesion features are extracted is very important, there are no clear cut answers on the best way to segment lesions. In this paper we opted for defining damaged voxels through lesion probability as a measure of atypicality regarding the expected tissues in healthy controls (Seghier et al., 2008), but there are other ways to define lesions. An interesting extension of this work would be to replicate the findings with other automatic segmentation approach and ultimately with manual tracing of lesions.

In summary, this study provides a thorough comparison of approaches to extract features based on patterns of voxels and lesion load to decode post-stroke impairment. It is also the first attempt to use multiple kernel learning to investigate the relevance of regions in predicting a score based on structural images from injured brain. Although our investigation is based on a cross-sectional analysis to ask whether current motor impairment can be explained by lesion characteristics, it illustrates why it is important to think about the methodology that one might use to predict long term outcome from imaging data acquired in the early stages after stroke. With respect to structural images we expect that data extracted from lesion characteristics will not change substantially over time, but this assumption needs to be tested in a prospective study.

## Figures and Tables

**Table 1:** Predicting motor impairment based on different masks to limit subsets of voxels. NF = number of features; R = correlation between real and predicted motor scores; RMSE = Root Mean Squared Error.

Model	Features	NF	R	RMSE
M1	Whole brain	630,786	0.72	0.73
M1.1	Voxels limited by AAL atlas	451,318	0.72	0.73
M1.2	Voxels limited by the corticospinal tract (CST)	4,421	0.65	0.75
M1.3	Voxels limited by AAL atlas + CST	457,384	0.73	0.72
M1.4	Voxels limited by motor ROIs	120,793	0.80	0.70
M1.5	Voxels limited by motor ROIs and CST	125,214	0.83	0.68
M1.6	Voxels limited by mask from task fMRI in healthy controls	35,545	0.67	0.78
M1.7	Voxels limited by lesion-symptom mapping	9991.1^a^	0.66	0.76
M1.8	Voxels limited by lesion in at least 1 patient	158,907	0.68	0.75

a Average across cross-validation folds.

**Table 2:** Predicting motor impairment based on different labelled ROIs to extract features by summarization of regions (lesion load). NF = number of features; R = correlation between real and predicted motor scores; RMSE = Mean Squared Error.

Model	Features	NF	R	RMSE
M2	Lesion load in the whole brain	1	0.30	0.94
M2.1	Lesion load in ROIs from AAL atlas	116	0.20	8.04
M2.2	Lesion load in corticospinal tract	1	0.51	0.84
M2.3	Lesion load in ROIs from AAL atlas + CST	117	0.25	6.47
M2.4	Lesion load in motor ROIs	22	0.21	1.10
M2.5	Lesion load in motor ROIs + CST	23	0.26	1.09
M2.6	Lesion load in functional mask from task fMRI	1	0.17	0.95
M2.7	Lesion load in ROIs defined by lesion-symptom mapping (median PCA)	5	0.31	0.92
M2.8	Lesion load in ROI from lesion in at least 1 patient	1	0.30	0.94

## Appendix A Supplementary data

Supplementary material. Image 1
