# Supplementary material for: Decoding post-stroke motor function from structural brain imaging
Source: Neuroimage Clin. 2016 Aug 2;12:372–80. doi: 10.1016/j.nicl.2016.07.014 (PMC4995603; doi:10.1016/j.nicl.2016.07.014)
Supplement: Supplementary file 1 — Supplementary material. [file mmc1.docx]

**1. Characteristics of the sample**

Table S1 presents characteristics of each patient enrolled in the study. The quantitative summary of each characteristic is presented in the last row. Gender is summarized as the total number of females (F) and the affected hand is summarized as the number of patients with right hand impairment (R). The other characteristics are summarized with average (standard deviation) of the sample.

The location of the lesions in each patient is presented in the table S1 (that also shows the proportion of voxels considered to be part of the lesion in each ROI).

**Table S1. Demographic and clinical characteristics of the patients**

| **Patient ID** | **Gender** | **Age (yrs)** | **Affected hand** | **Time since stroke (months)** | **Lesion volume (voxels)** | **ARAT** | **Grip** | **MI** | **NHPT** |
| --- | --- | --- | --- | --- | --- | --- | --- | --- | --- |
| 1 | M | 60 | L | 6 | 3589 | 26 | 24.9 | 48 | 5.1 |
| 2 | M | 60 | L | 41 | 24326 | 39 | 20.1 | 65 | 0 |
| 3 | M | 48 | L | 116 | 11068 | 54 | 87.6 | 88 | 70.8 |
| 4 | M | 46 | R | 8 | 2070 | 57 | 106.2 | 100 | 104.5 |
| 5 | F | 62 | L | 3 | 3568 | 52 | 111.7 | 93 | 60.6 |
| 6 | M | 69 | R | 9 | 3599 | 57 | 80.5 | 100 | 69.7 |
| 7 | M | 44 | L | 8 | 5437 | 36 | 78.6 | 81 | 5.1 |
| 8 | M | 33 | R | 63 | 3048 | 57 | 71.7 | 100 | 68.9 |
| 9 | M | 51 | R | 4 | 569 | 57 | 64.2 | 100 | 89.6 |
| 10 | M | 48 | L | 7 | 27919 | 31 | 35.3 | 42 | 0 |
| 11 | M | 58 | L | 7 | 1270 | 57 | 93.3 | 100 | 74.5 |
| 12 | M | 59 | L | 3 | 38204 | 52 | 64.4 | 42 | 14.9 |
| 13 | M | 22 | R | 3 | 2448 | 57 | 93.1 | 100 | 94.5 |
| 14 | F | 69 | L | 30 | 4225 | 56 | 84 | 85 | 19.8 |
| 15 | M | 42 | L | 26 | 19753 | 44 | 80.6 | 85 | 0 |
| 16 | M | 70 | L | 3 | 9011 | 57 | 81.3 | 100 | 81.3 |
| 17 | F | 50 | L | 18 | 33415 | 28 | 41.3 | 66 | 8.2 |
| 18 | F | 40 | L | 21 | 15679 | 52 | 58 | 93 | 25.5 |
| 19 | M | 80 | L | 20 | 3858 | 26 | 56.3 | 74 | 4 |
| 20 | M | 75 | L | 6 | 6600 | 57 | 96.6 | 100 | 73.7 |
| 21 | F | 55 | L | 5 | 1320 | 55 | 64 | 93 | 97 |
| 22 | F | 53 | L | 31 | 1391 | 50 | 40 | 91 | 50 |
| 23 | F | 59 | L | 165 | 6210 | 29 | 18.2 | 68 | 0 |
| 24 | F | 77 | R | 26 | 7332 | 38 | 57.2 | 77 | 9 |
| 25 | M | 58 | L | 11 | 39409 | 57 | 88.2 | 100 | 87 |
| 26 | F | 56 | R | 16 | 5486 | 36 | 44 | 77 | 9 |
| 27 | M | 62 | L | 38 | 4325 | 36 | 31 | 72 | 8 |
| 28 | M | 59 | L | 79 | 24482 | 21 | 50.3 | 73 | 0 |
| 29 | M | 63 | R | 8 | 4459 | 57 | 91 | 100 | 77.9 |
| 30 | M | 58 | L | 13 | 16587 | 37 | 66 | 64 | 9 |
| 31 | M | 66 | R | 26 | 38714 | 35 | 81 | 65 | 39 |
| 32 | M | 53 | R | 12 | 8653 | 48 | 52 | 91 | 53 |
| 33 | F | 43 | R | 20 | 13736 | 41 | 71 | 91 | 31 |
| 34 | M | 36 | R | 20 | 4745 | 54 | 81.9 | 93 | 31 |
| 35 | F | 45 | L | 29 | 6450 | 45 | 51 | 85 | 35 |
| 36 | F | 52 | R | 83 | 42304 | 27 | 31 | 73 | 0 |
| 37 | M | 51 | R | 60 | 6778 | 45 | 104 | 92 | 31 |
| 38 | M | 61 | L | 13 | 2226 | 45 | 51.1 | 65 | 19.7 |
| 39 | F | 42 | L | 22 | 25769 | 45 | 65 | 91 | 21 |
| 40 | F | 56 | R | 32 | 8526 | 39 | 41 | 77 | 9 |
| 41 | M | 57 | R | 17 | 10528 | 57 | 84 | 84 | 71 |
| 42 | M | 52 | L | 24 | 3579 | 42 | 35 | 88 | 9 |
| 43 | M | 50 | R | 13 | 7744 | 57 | 68 | 100 | 60 |
| 44 | F | 69 | L | 38 | 6690 | 57 | 94 | 93 | 50 |
| 45 | F | 40 | L | 25 | 16720 | 55 | 60 | 93 | 30 |
| 46 | M | 46 | L | 26 | 8461 | 46 | 52.3 | 84 | 38 |
| 47 | M | 56 | R | 13 | 22623 | 37 | 107 | 91 | 40 |
| 48 | M | 66 | L | 76 | 6233 | 21 | 46.6 | 72 | 0 |
| 49 | F | 18 | L | 5 | 14037 | 44 | 27.7 | 93 | 5.9 |
| 50 | M | 66 | L | 5 | 5409 | 57 | 63.4 | 92.5 | 98.2 |
| **Summary** | **17 F** | **54.22**  **(12.62)** | **18 R** | **27.06**  **(31.05)** | **11811.64**  **(11365.58)** | **45.30**  **(11.28)** | **64.93**  **(24.58)** | **83.81**  **(15.30)** | **37.78**  **(33.13)** |

The figure S1 presents a matrix showing the proportion of each region affected by lesions in each patient. Each column (from 1 to 50) relates to a particular patient and each row corresponds to an ROI from the AAL atlas. Only ROIs that were affected in at least one patient are presented. The colour scale (from 0 to 1) represents the lesion load (i.e., the number of voxels in the ROI considered to be part of the lesion divided by the ROI size).

Figure S1. Lesion load per ROI for each patient

**2. Lesion segmentation**

We evaluated whether the filter to eliminate small clusters of contiguous voxels in the binary images resulting from segmentation was necessary in our sample. Given that stroke lesions typically involve neighbouring voxels, a considerable amount of single, false-positive voxels would not be expected. However, we checked the segmentation of each patient and noticed that the filter was indeed necessary in our images, as suggested by (Seghier et al., 2008). The figure S2 illustrates an example, where false positive voxels (represented in red) were above the threshold, but they formed small clusters with less than 100 voxels, thus being excluded. Additionally, in order to check whether this filter is not eliminating small lesions, we checked the segmentation of all patients. The smallest lesion is a contiguous volume with 569 voxels.

**Figure S2. Example of segmentation without filtering small clusters (represented in red)**

Regarding the threshold level, we did segmentations with alternative values slightly below and above 0.30 (the level suggested by (Seghier et al., 2008)) and repeated the regression analysis, whose results are presented in Table S2 (threshold 0.25) and Table S3Table S3 (threshold 0.35). These results did not show systematic improvement regarding results from analysis using the suggested threshold (presented in the manuscript, table 3).

**Table S2. Prediction of motor scores based on lesion load segmented with threshold 0.25**

| **Model** | **Features** | **NF** | **R** | **MSE** |
| --- | --- | --- | --- | --- |
| M2 | Lesion load in the whole brain | 1 | 0.28 | 0.95 |
| M2.1 | Lesion load in ROIs from AAL atlas | 116 | 0.31 | 2.27 |
| M2.2 | Lesion load in Corticospinal tract | 1 | 0.50 | 0.84 |
| M2.3 | Lesion load in ROIs from AAL atlas + CST | 117 | 0.23 | 1.87 |
| M2.4 | Lesion load in motor ROIs | 22 | 0.32 | 3.48 |
| M2.5 | Lesion load in motor ROIs + CST | 23 | 0.31 | 3.60 |
| M2.6 | Lesion load in Functional mask from task fMRI | 1 | 0.25 | 0.92 |
| M2.7 | Lesion load in ROIs defined by Lesion-symptom mapping | 5 | 0.30 | 0.94 |
| M2.8 | Lesion load in ROI from lesion in at least 1 patient | 1 | 0.29 | 0.94 |

**Table S3. Prediction of motor scores based on lesion load segmented with threshold 0.35**

| **Model** | **Features** | **NF** | **R** | **MSE** |
| --- | --- | --- | --- | --- |
| M2 | Lesion load in the whole brain | 1 | 0.30 | 0.93 |
| M2.1 | Lesion load in ROIs from AAL atlas | 116 | 0.10 | 120.95 |
| M2.2 | Lesion load in Corticospinal tract | 1 | 0.49 | 0.84 |
| M2.3 | Lesion load in ROIs from AAL atlas + CST | 117 | 0.29 | 182.10 |
| M2.4 | Lesion load in motor ROIs | 22 | 0.11 | 1.14 |
| M2.5 | Lesion load in motor ROIs + CST | 23 | 0.16 | 1.13 |
| M2.6 | Lesion load in Functional mask from task fMRI | 1 | 0.25 | 0.91 |
| M2.7 | Lesion load in ROIs defined by Lesion-symptom mapping | 5 | 0.30 | 0.92 |
| M2.8 | Lesion load in ROI from lesion in at least 1 patient | 1 | 0.30 | 0.93 |

**3. Support vector regression**

The results of the application of SVR with the same kernel are presented in the tables S4 and S5.

**Table S4. SVR prediction of motor scores based on patterns of voxels representing lesion probability**

| **Model** | **Features** | **NF** | **R** | **MSE** |
| --- | --- | --- | --- | --- |
| M1 | Whole brain | 630786 | 0.67 | 0.61 |
| M1.1 | Voxels limited by AAL atlas | 451318 | 0.69 | 0.59 |
| M1.2 | Voxels limited by the Corticospinal tract (CST) | 4421 | 0.56 | 0.67 |
| M1.3 | Voxels limited by AAL atlas + CST | 457384 | 0.70 | 0.58 |
| M1.4 | Voxels limited by motor ROIs | 120793 | 0.74 | 0.57 |
| M1.5 | Voxels limited by motor ROIs and CST | 125214 | 0.77 | 0.54 |
| M1.6 | Voxels limited by mask from task fMRI in healthy controls | 35545 | 0.67 | 0.72 |
| M1.7 | Voxels limited by lesion-symptom mapping | ** 9991.1* | 0.57 | 0.62 |
| M1.8 | Voxels limited by lesion in at least 1 patient | 158907 | 0.66 | 0.57 |

**Table S5. SVR prediction of motor scores based on lesion load**

| **Model** | **Features** | **NF** | **R** | **MSE** |
| --- | --- | --- | --- | --- |
| M2 | Lesion load in the whole brain | 1 | 0.29 | 1.02 |
| M2.1 | Lesion load in ROIs from AAL atlas | 116 | 0.47 | 0.91 |
| M2.2 | Lesion load in Corticospinal tract | 1 | 0.50 | 0.86 |
| M2.3 | Lesion load in ROIs from AAL atlas + CST | 117 | 0.43 | 0.92 |
| M2.4 | Lesion load in motor ROIs | 22 | 0.36 | 1.23 |
| M2.5 | Lesion load in motor ROIs + CST | 23 | 0.37 | 1.29 |
| M2.6 | Lesion load in Functional mask from task fMRI | 1 | 0.17 | 0.95 |
| M2.7 | Lesion load in ROIs defined by Lesion-symptom mapping | 5 | 0.40 | 0.86 |
| M2.8 | Lesion load in ROI from lesion in at least 1 patient | 1 | 0.29 | 1.02 |

**4. Multiple kernel learning applied to ROIs**

Table S6 presents a list of all ROIs (116 areas from the AAL atlas and the CST divided into left and right portions). The ROIs are ranked in descending order according to the weight assigned to them by the multiple kernel learning analysis. The figure S3 gives an intuition of the distribution of the weights across the ROIs through a bar graph.

**Table S6. Rois sorted by weight in multiple kernel learning analysis**

| **Ranking** | **ROI name** | **Weight** |
| --- | --- | --- |
| 1 | Right caudate nucleus | 0.0267 |
| 2 | Left inferior frontal gyrus, pars opercularis | 0.0264 |
| 3 | Right corticospinal tract | 0.0262 |
| 4 | Left middle temporal pole | 0.0259 |
| 5 | Right lobule 7b of cerebellar hemisphere | 0.0256 |
| 6 | Right superior temporal pole | 0.0247 |
| 7 | Right lobule 9 of cerebellar hemisphere | 0.0239 |
| 8 | Left Cuneus | 0.0236 |
| 9 | Left caudate nucleus | 0.0233 |
| 10 | Lobule VII of vermis | 0.0231 |
| 11 | Right lobule 10 of cerebellar hemisphere | 0.0230 |
| 12 | Left calcarine sulcus | 0.0227 |
| 13 | Lobule VI of vermis | 0.0226 |
| 14 | Lobule VIII of vermis | 0.0223 |
| 15 | Right cuneus | 0.0223 |
| 16 | Left globus pallidus | 0.0222 |
| 17 | Right crus II of cerebellar hemisphere | 0.0218 |
| 18 | Lobule IV and V of vermis | 0.0216 |
| 19 | Left middle frontal gyrus, orbital part | 0.0214 |
| 20 | Right Amygdala | 0.0209 |
| 21 | Left medial frontal gyrus | 0.0208 |
| 22 | Right Hippocampus | 0.0207 |
| 23 | Left lobule 7b of cerebellar hemisphere | 0.0201 |
| 24 | Lobule X of vermis | 0.0200 |
| 25 | Left inferior temporal gyrus | 0.0191 |
| 26 | Right rectus gyrus | 0.0189 |
| 27 | Left lobule 4_5 of cerebellar hemisphere | 0.0188 |
| 28 | Left transverse temporal gyrus | 0.0186 |
| 29 | Right globus pallidus | 0.0184 |
| 30 | Left superior temporal gyrus | 0.0183 |
| 31 | Right middle occipital gyrus | 0.0183 |
| 32 | Lobule I and II of vermis | 0.0182 |
| 33 | Left superior parietal lobule | 0.0180 |
| 34 | Left supplementary motor area | 0.0180 |
| 35 | Right paracentral lobule | 0.0180 |
| 36 | Left putamen | 0.0179 |
| 37 | Left corticospinal tract | 0.0178 |
| 38 | Left angular gyrus | 0.0178 |
| 39 | Right superior temporal gyrus | 0.0177 |
| 40 | Right inferior frontal gyrus, pars opercularis | 0.0172 |
| 41 | Left olfactory cortex | 0.0171 |
| 42 | Right lobule 8 of cerebellar hemisphere | 0.0167 |
| 43 | Left fusiform gyrus | 0.0166 |
| 44 | Left lingual gyrus | 0.0165 |
| 45 | Left superior occipital | 0.0165 |
| 46 | Right superior occipital | 0.0165 |
| 47 | Right ParaHippocampal gyrus | 0.0164 |
| 48 | Right inferior frontal gyrus, pars triangularis | 0.0164 |
| 49 | Left Rolandic operculum | 0.0164 |
| 50 | Right Rolandic operculum | 0.0162 |
| 51 | Right precentral gyrus | 0.0161 |
| 52 | Left Thalamus | 0.0160 |
| 53 | Right supramarginal gyrus | 0.0159 |
| 54 | Left supramarginal gyrus | 0.0159 |
| 55 | Right inferior temporal gyrus | 0.0158 |
| 56 | Left lobule 10 of cerebellar hemisphere | 0.0156 |
| 57 | Left precuneus | 0.0154 |
| 58 | Left gyrus rectus | 0.0154 |
| 59 | Right inferior parietal lobule | 0.0152 |
| 60 | Right Fusiform gyrus | 0.0152 |
| 61 | Right posterior cingulate gyrus | 0.0151 |
| 62 | Right postcentral gyrus | 0.0148 |
| 63 | Left lobule 3 of cerebellar hemisphere | 0.0147 |
| 64 | Right middle frontal gyrus, orbital part | 0.0146 |
| 65 | Left lobule 9 of cerebellar hemisphere | 0.0146 |
| 66 | Right lobule 4_5 of cerebellar hemisphere | 0.0145 |
| 67 | Left crus I of cerebellar hemisphere | 0.0144 |
| 68 | Right crus I of cerebellar hemisphere | 0.0143 |
| 69 | Right Thalamus | 0.0143 |
| 70 | Right supplementary motor area | 0.0141 |
| 71 | Left precentral gyrus | 0.0140 |
| 72 | Left superior frontal gyrus, orbital part | 0.0140 |
| 73 | Right inferior occipital cortex | 0.0139 |
| 74 | Right inferior frontal gyrus, pars orbitalis | 0.0139 |
| 75 | Right calcarine sulcus | 0.0136 |
| 76 | Right middle temporal pole | 0.0135 |
| 77 | Right insula | 0.0133 |
| 78 | Right Putamen | 0.0133 |
| 79 | Right transverse temporal gyrus | 0.0132 |
| 80 | Left medial frontal gyrus | 0.0132 |
| 81 | Left lobule 8 of cerebellar hemisphere | 0.0130 |
| 82 | Left ParaHippocampal gyrus | 0.0129 |
| 83 | Right lingual gyrus | 0.0129 |
| 84 | Left superior temporal pole | 0.0128 |
| 85 | Left Hippocampus | 0.0127 |
| 86 | Right lobule 6 of cerebellar hemisphere | 0.0127 |
| 87 | Left Amygdala | 0.0126 |
| 88 | Right superior frontal gyrus | 0.0124 |
| 89 | Lobule IX of vermis | 0.0124 |
| 90 | Right middle temporal gyrus | 0.0124 |
| 91 | Left inferior occipital cortex | 0.0120 |
| 92 | Left superior frontal gyrus | 0.0120 |
| 93 | Right superior parietal lobule | 0.0118 |
| 94 | Left lobule 6 of cerebellar hemisphere | 0.0117 |
| 95 | Right olfactory cortex | 0.0116 |
| 96 | Left middle occipital gyrus | 0.0116 |
| 97 | Left midcingulate area | 0.0116 |
| 98 | Left middle temporal gyrus | 0.0113 |
| 99 | Right lobule 3 of cerebellar hemisphere | 0.0113 |
| 100 | Right angular gyrus | 0.0112 |
| 101 | Left middle frontal gyrus, orbital part | 0.0111 |
| 102 | Left postcentral gyrus | 0.0110 |
| 103 | Lobule III of vermis | 0.0108 |
| 104 | Left crus II of cerebellar hemisphere | 0.0107 |
| 105 | Right middle frontal gyrus, orbital part | 0.0104 |
| 106 | Left paracentral lobule | 0.0103 |
| 107 | Right precuneus | 0.0103 |
| 108 | Right anterior cingulate gyrus | 0.0099 |
| 109 | Right medial frontal gyrus | 0.0099 |
| 110 | Right midcingulate area | 0.0098 |
| 111 | Right superior frontal gyrus, orbital part | 0.0097 |
| 112 | Right middle frontal gyrus | 0.0097 |
| 113 | Left inferior frontal gyrus, pars orbitalis | 0.0096 |
| 114 | Left insula | 0.0095 |
| 115 | Left anterior cingulate gyrus | 0.0094 |
| 116 | Left inferior parietal lobule | 0.0087 |
| 117 | Left posterior cingulate gyrus | 0.0083 |
| 118 | Left inferior frontal gyrus, pars triangularis | 0.0081 |

**Figure S3 Weight of each pattern ROI to predict motor impairment using multiple kernel learning**

**5. Number of features and performance**

In order to investigate how the number of features used relates to the performance of the regression algorithm independent of the anatomical information, we selected a subset of ROIs from the AAL atlas in a random way. ROIs were randomly selected until the total number of voxels reached the order of the model M1.4 (table 1, 120793 voxels). Using the pattern of voxels delimited by this mask and applying GPR to predict the motor score, the correlation between real and predicted labels was R = 0.61; MSE = 0.77. The accuracy was lower than using the pattern of voxels delimited by both the whole brain (model M1: R = 0.72; MSE = 0.73) and the motor ROIs (model M1.4: R=0.80; MSE = 0.70). This result suggests that the influence of the number of features in the performance is not independent of the anatomical information.
